# Comparison of tacrolimus with or without prednisone therapy in primary membranous nephropathy: a retrospective clinical study

**DOI:** 10.1038/s41598-024-64661-w

**Published:** 2024-06-20

**Authors:** Xinyue Zhang, Jingyu Dou, Ge Gao, Xiaoxiao Sheng, Ya Shen, Yuhua Feng, Xueying Wu, Zhen Zhang, Genyang Cheng

**Affiliations:** Department of Nephrology, The First Affiliated Hospital of Zhengzhou University, Zhengzhou, China

**Keywords:** Primary membranous nephropathy, Tacrolimus, Prednisone, Therapy, Relapse, Immunology, Nephrology

## Abstract

Previous studies showed tacrolimus monotherapy and dual therapy with tacrolimus and prednisone as effective treatment modalities in managing membranous nephropathy. However, few studies have compared these therapeutic regimens. The patients were divided into two groups based on the treatment regimen: (1) tacrolimus and prednisone dual therapy (T + P group, n = 67) treatment group; and (2) tacrolimus monotherapy (T group, n = 65) or the control group. Propensity matching method and subgroup analysis to eliminate the bias in the relationship between the treatment regimen and the outcomes. The mean remission times were 20.33 ± 2.75 weeks at T group and 9.50 ± 1.81 weeks at T + P group. The T group had a remission rates of 73.33, 76.66 and 66.66% at 12weeks, 24weeks and 48weeks, while the T + P group had a remission rate of 81.66, 86.66, 91.66%; At the follow-up of 48 weeks, the relapse rate for the T group was 21.66%, and that for the T + P group was 5%. The anti-PLA2R ab is positive and therapy may be the independent risk factors for predicting remission. Tacrolimus and low-dose prednisone dual therapy is efficacious in managing MN and lowers the recurrence rate in clinical practice.

## Introduction

Membranous nephropathy (MN) is a chronic disease that affects individuals of all ages and races. However, the treatment of MN remains challenging. The pathological manifestations of primary membranous nephropathy (PMN) include capillary wall thickening, normal cellularity, IgG and C3 deposits along the capillary walls that are detected using immunofluorescence, and subepithelial deposits observed under electron microscopy^1^. The diagnostic marker for MN is the presence of antibodies against the phospholipase A2 receptor (PLA2R) which are initially detected in the serum of 70% of patients with MN^2^. Additionally, previous studies revealed that thrombospondin type-1 domain-containing 7A antibodies are present in most patients with MN^3,4^. The clinical course of MN varies, and approximately 30% of patients with nephrotic syndrome experience spontaneous remission. The remaining patients may experience slow progression, relapse, or develop proteinuria^5^. The Kidney Disease: Improving Global Outcomes clinical practice guidelines^6^ recommend the use of rituximab and tacrolimus ± glucocorticoids for patients with moderate-risk PMN. Rituximab has demonstrated safety and efficacy in inducing immunological and clinical remission in patients with MN. However, rituximab is not covered by health insurance in China as an initial treatment for PMN. Cyclophosphamide is the standard treatment for intermediate- or high-risk PMN. However, many physicians and patients are reluctant to use it due its association with an increased risk of cancer^7^. Notably, the risk of cyclophosphamide-related bladder cancer is dose-dependent^8,9^.

Tacrolimus (TAC, FK506) is a calmodulin nerve phosphatase inhibitor widely used in clinical management of organ transplantation and the treatment of autoimmune diseases^10,11^. A prospective study demonstrated that TAC monotherapy provided a high remission rate of 94% with no relapse during the treatment period^12^. Caro et al. and Ballarin et al. observed that TAC monotherapy effectively treated MN. However, patients with partial remission (PR) frequently relapsed^13^. A study by Yuan et al. reported that combining TAC with low-dose prednisone markedly improved MN with a remission rate of 90% at 6 months, and prolonged TAC use significantly reduced MN recurrence rates^14^. Another study^15^ showed that TAC effectively treated mild mesangial proliferative glomerulonephritis when used in combination with corticosteroids.

Although previous studies have indicated that TAC is effective in treating PMN, the relapse rate is high. Moreover, few studies have compared TAC monotherapy and TAC plus low-dose prednisone dual therapy for the treatment of MN. Therefore, we aimed to investigate the efficacy, safety, and relapse rates of these treatment regimens for managing PMN.

## Material and methods

### Patients

The pathological and pharmacological records of patients with PMN who were treated at our center from January 2015 to June 2019 were retrieved and analyzed. A total of 132 patients who were treated with TAC and low-dose prednisone dual therapy (T + P), or TAC monotherapy (T) were selected. Nephrologists selected the treatment regimens based on the treatment guidelines that determine the risk of femoral head necrosis. The patients were divided into two groups according to the selected treatment regimen. The inclusion criteria were as follows: (1) age 18–80 years, (2) nephrotic syndrome, and (3) pathology consistent with PMN. The exclusion criteria were as follows: (1) MN secondary to hepatitis B virus infection, malignancy, or systemic lupus erythematosus; (2) patients without regular follow-up and < 12 weeks follow-up; and (3) treatment with or switching to additional immunosuppressive drugs (e.g., cyclophosphamide, mycophenolate mofetil). The ethical review board of the First Affiliated Hospital of Zhengzhou University approved this retrospective chart analysis. Written informed consent was obtained from all participants. All methods used in this study were based on relevant guidelines and regulations, and patient identifiers were not obtained during or after data collection. Baseline clinical data were collected at the time of renal biopsy, and anti-PLA2Rab levels, 24-h urine proteinuria levels, TAC trough blood levels, serum albumin (ALB), serum creatinine, and estimated glomerular filtration rates were measured until the end of the follow-up period (September 2020).

### Therapeutic regimen

The 2021 KDIGO guidelines recommend the use of TAC to treat MN. In a placebo-controlled randomized trial, TAC monotherapy was more effective than immunosuppressant therapy for patients with MN^16^. The PMN treatment regimen consisted of an initial oral dose of 0.05 mg/kg/d TAC, with trough blood level maintained at 5–10 ng/mL. The serum TAC concentration was assessed and the dose adjusted accordingly. Patients achieved complete remission (CR) or partial remission (PR) after 2 months, and the TAC dose was gradually reduced. The dose was reduced by 25% on a monthly basis, and serum levels of TAC were routinely reviewed and maintained at 3–6 ng/mL. Oral prednisone was initiated at 0.5 mg/kg/d and increased as needed to a maximum dose of 30 mg/day. When patients achieved CR after 2 weeks, the dose was reduced by 5 mg biweekly. Approximately four weeks later, the patients were instructed to reduce the dosage by 2.5 mg biweekly until a final dose of 10 mg/day was achieved before discontinuation. During the follow-up period, patients in the T + P and T groups were treated with angiotensin-converting enzyme inhibitors or angiotensin receptor blockers and blood pressure was maintained at < 130/80 mmHg. Almost all patients presented with nephrotic syndrome and received statins and anticoagulants at the initial treatment.

### Definitions

Various studies on immunosuppressive therapy in Chinese adults with nephrotic syndrome have demonstrated that patients with nephrotic syndrome exhibit marked proteinuria (24 h urine proteinuria > 3.5g), hypoproteinemia (ALB < 30 g/L), edema, and hyperlipidemia. The following criteria were used to determine CR: a 24-h urine protein < 0.3 g or urine protein/creatinine level (< 300 mg/g), normal renal function, and normal serum ALB. PR was described as follows: a 24-h urine protein within the range of 0.3–3.5 g, a urine protein/creatinine level of 300–3,500 mg/g, or a 24 h urine protein reduced by 50% of the baseline. Relapse was defined as the recurrence of severe proteinuria or a urine protein/creatinine > 3,500 mg/g after the patient entered remission.

### Evaluation and outcome measures

Clinical and histological data, including sex, age, 24 h urine proteinuria, ALB level, and kidney biopsy chronicity score (by Sethi et al., S Table 1) were retrieved from patient records. Weekly monitored parameters included ALB (g/L), serum creatinine (Scr, µmol/L), anti-PLA2Rab (RU/mL, using the ELISA test; level > 14 RU/mL was considered positive)^17^, 24 h proteinuria (g), and estimated glomerular filtration rate (eGFR, mL/min/1.73 m^2^) using the CKD-Epidemiology Collaboration (CKD-EPI) formula^18^. The numbers of patients with CR, PR, relapse, and complications were evaluated at weeks 2, 4, 8, 12, 24, 36, and 48.

### Statistical analysis

IBM SPSS software (IBM Corp. Released 2017. IBM SPSS Statistics for Windows, Version 25.0. Armonk, NY: IBM Corp.) was used for statistical analyses, and GraphPad Prism 8.00 (GraphPad Software, Inc., USA) was used for the multivariable analyses. Quantitative variables are expressed as medians and interquartile ranges. Qualitative variables are presented as percentages. Non-parametric variables were compared using the Mann–Whitney U test. The propensity score matching (PSM) method was used at a 1:1 ratio, and the matching variables were serum albumin level, proteinuria, eGFR, and anti-PLA2Rab. Matching was performed with nearest-neighbor and without replacement, and the caliper distance was 0.02. The cumulative probabilities of remission were assessed using Kaplan–Meier survival analysis and the log-rank test. Time represents the period from baseline to when remission was first achieved. The independent predictors of remission were screened using COX regression analysis. Significant variables in univariate analysis were included in multivariate COX regression analysis. Statistical significance was set at P < 0.05 significant.

## Results

### Baseline clinical characteristics

**Figure 1:**
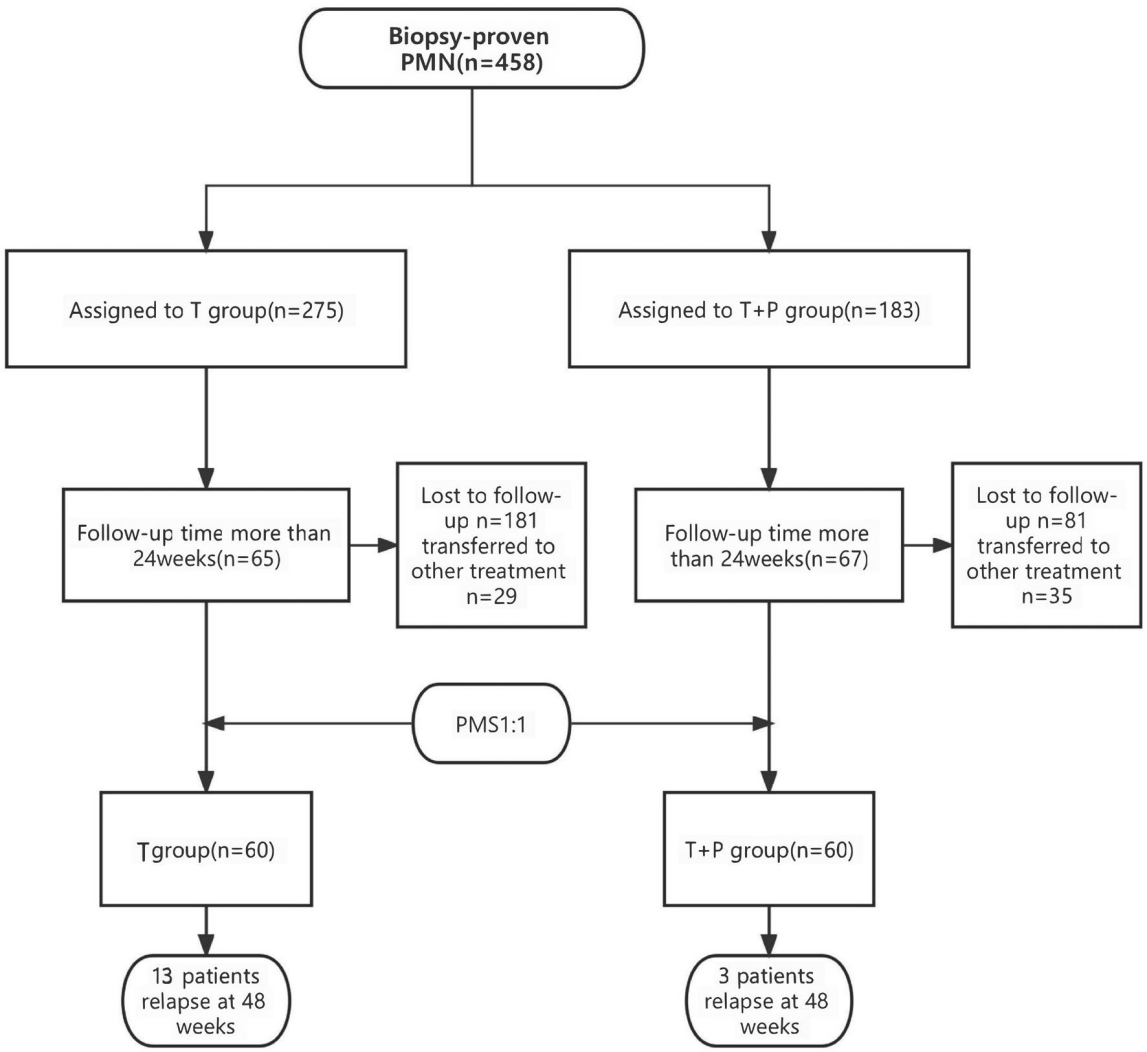
Group allotment. Enrolment of patients, treatment assignments and number in each group.

One hundred and thirty-two patients diagnosed with MN by biopsy were identified (Fig. 1). Of these patients, 67 (women: 28; men: 39) were assigned to the T + P group and received TAC and prednisone dual therapy (Table 1). The remaining 65 patients who received TAC monotherapy, were assigned to the control (T) group. After PSM was performed, a total of 60 pairs of patients were successfully matched. The baseline patient characteristics are presented in Table 2. There were no significant differences in baseline clinical and laboratory characteristics between the groups before and after matching.

**Table 1:** Characteristics of patients in the two study groups before matched.

	T group (n=65)	T+P group (n=67)	Z/χ^2^	P value
Gender (male/female)	30/35	39/28	1.922	0.166
Age (years old)	47(34,57)	50(45,63)	−0.118	0.906
SBP (mmHg)	127.5(119.3,138.8)	130.0(123.0,138.0)	−0.371	0.711
DBP (mmHg)	80.0(75.3,89.3)	85.0(78.0,90.0)	−1.568	0.117
Hb (g/L)	126.9(115.0,133.0)	131.8(116.0,143.0)	−1.451	0.147
ALB (g/L)	26.3(22.3,28.6)	24.6(22.1,28.0)	−0.835	0.404
Scr (umol/L)	63(53.5,76.0)	67.0(59.0,78.0)	−1.248	0.212
eGFR (mL/min/1.73 m^2^)	106.3(96.8,112.4)	101.8(89.6,110.8)	−0.048	0.962
PLA2Rab(Ru/mL)	65.1(17.3,162.2)	42.8(14.0,158.9)	−0.021	0.983
High titer/non-high titer	21/44	19/48	0.244	0.622
TC (mmol/L)	7.0(5.7,9.0)	6.8(5.3,8.4)	−0.035	0.972
TG (mmol/L)	1.9(1.4,3.1)	2.1(1.5,2.6)	−0.327	0.743
Blood-glucose (mmol/L)	4.6(4.2,5.0)	4.6(4.2,4.9)	−1.139	0.255
24-hour urine proteinuria (g/24h)	3.9(3.6,6.0)	4.9(3.6,6.8)	−0.521	0.602
Chronicity score	2(1,3)	2(1,3)	0.447	0.932

**Table 2:** Characteristics of patients in the two study groups after matched.

	T group (n=60)	T+P group (n=60)	Z/χ2	P value
Gender (male/female)	29/31	34/26	0.020	0.888
Age (years old)	46.5(33.5,57.5)	47(39,54.7)	−0.071	0.943
SBP (mmHg)	130(119,139)	129(123,136)	−0.202	0.840
DBP (mmHg)	80.0(75.0,90.0)	83.5(78.0,90.0)	−1.418	0.156
Hb (g/L)	126.5(116.5,133.0)	133(114.0,143.0)	−1.674	0.094
ALB (g/L)	26.2(22.0,28.4)	25.1(22.3,28.3)	−0.129	0.898
Scr (umol/L)	63(55,75.0)	62.0(57.2,75.7)	−0.714	0.475
eGFR (mL/min/1.73 m^2^)	104.4(96.2,112.4)	105.0(95.2,117.4)	−0.346	0.729
PLA2Rab(Ru/mL)	63.2(19.4,162.8)	60.8(20.4,144.6)	−0.069	0.490
High titer/non-high titer	19/41	15/45	0.657	0.418
TC (mmol/L)	6.9(4.0,8.2)	6.8(5.4,9.0)	−0.062	0.951
TG (mmol/L)	1.9(1.4,3.1)	2.1(1.3,3.1)	−0.060	0.952
Blood-glucose (mmol/L)	4.6(4.2,4.9)	4.5(4.2,4.9)	−0.959	0.337
24-hour urine proteinuria (g/24h)	3.9(3.6,5.8)	4.5(3.6,6.6)	−0.436	0.663
Chronicity score	2(1,3)	2(1,2)	0.456	0.985

### Changes in 24-h proteinuria, albumin level, serum creatinine, and PLA2Rab levels in both groups during the follow-up period in both groups

**Figure 2:**
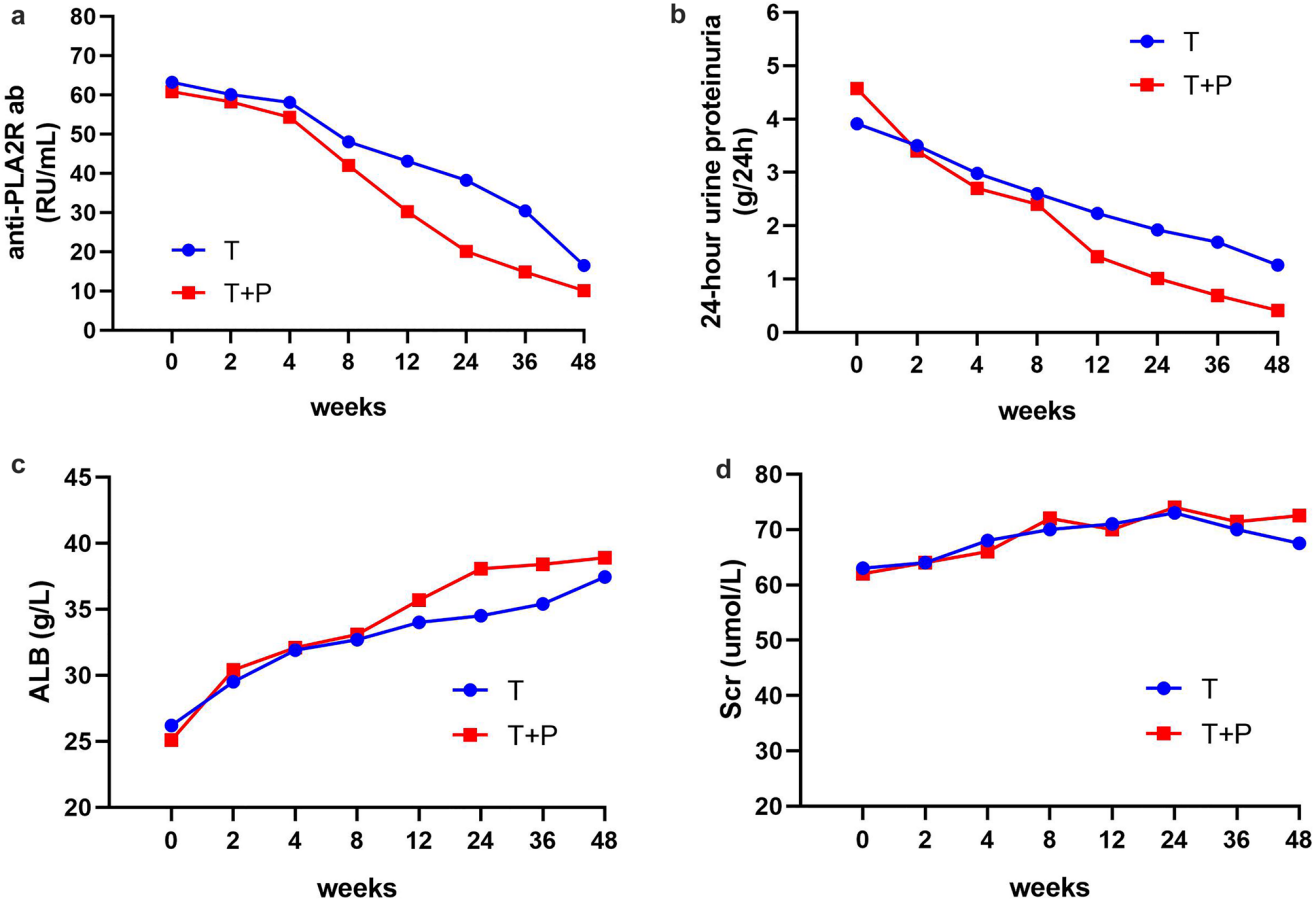
(**a**) PLA2Rab level in the two groups. During entire follow-up, anti-PLA2Rab in T group was higher than that in T + P group, and at 8 weeks, the T + P group decreased rapidly. There was significant difference between the two groups at 48 weeks. (**b**) 24 h urine proteinuria level in the two groups. There was a decrease in 24 h urine proteinuria in both T and T + P group. After 12 weeks, the decrease in urinary protein was more in T + P group than the T group, at 48 weeks, there was significant difference between the two groups. (**c**) Serum albumin level in the two groups. Serum albumin increased in both T and T + P groups. After 12 weeks, the increasing trend of serum albumin in T + P group was slightly higher than that in T group, but there was no significant difference between the two groups. (**d**) Serum creatinine level in the two groups. During entire follow-up, serum creatinine in T + P group was the same in T group, and there was no significant difference between the two groups.

During the follow-up period, anti-PLA2Rab levels (Table 3; Fig. 2a), and the 24 h urinary protein levels (Table 3; Fig. 2b) decreased in both groups. At 48 weeks, the median 24-h urinary protein of the T + P group decreased to 0.41 g and the median PLA2Rab level had decreased to 10.1 RU/mL. In the T group, the median 24 h urinary protein decreased to 1.26 g and the median PLA2Rab level decreased to 16.5 RU/mL. There were significant differences in the 24-h urinary protein (P = 0.010) and PLA2Rab levels (P = 0.024) between the groups.

**Table 3:** 24-h urine proteinuria, albumin level, estimated glomerular filtration rates, serum creatinine level and PLA2Rab level of patients in the two groups at 48 weeks.

	T group (n = 60)	T + P group (n = 60)	Z	P value
Scr (umol/L)	67.5(57.2,81.7)	72.5(63.2,82.0)	−0.940	0.347
eGFR (mL/min/1.73 m^2^)	99.7(84.3,114.2)	100.2(86.5,111.0)	−0.194	0.846
PLA2Rab(RU/mL)	16.5(2.7,31.7)	10.1(2.1,18.2)	−2.254	0.024
ALB (g/L)	37.4(31.4,40.8)	38.9(32.9,44.2)	−1.722	0.085
24-h urine proteinuria (g/24h)	1.26(0.24,4.48)	0.41(0.14,1.88)	−2.577	0.010

The ALB (Table 3; Fig. 2c) and Scr levels (Table 3; Fig. 2d) gradually increased. Statistical analyses revealed no significant differences between the groups.

### Subgroup analysis were performed according to the anti-PLA2R ab titers

Recent research suggests that antibody titers may impact the prognosis and reflect the degree of disease activity^19,20^. Subgroup analysis was conducted based on the anti-PLA2Rab titers, which were divided into the anti-PLA2Rab-positive (level ≥ 14 RU/mL, n = 107) and anti-PLA2Rab-negative (level < 14 RU/mL, n = 13) groups (S Table 2), high titer (> 150 RU/mL, n = 34) and non-high titer (2–150 RU/mL, n = 86) groups (S Table 3). Significant differences were identified in the 24 h urinary protein levels between the two groups but not in the ALB and creatinine levels (S Table 2). In the anti-PLA2Rab-positive group, the T + P group exhibited a higher remission rate.

### Remission and relapse rates

**Figure 3:**
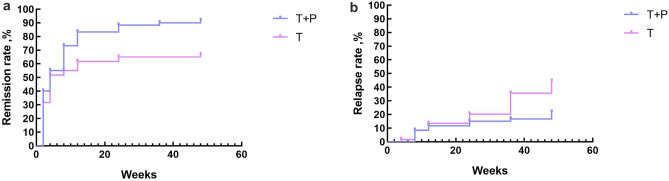
(**a**) Differences in the remission rate. There was a significant difference between the two groups (P = 0.001). (**b**) Differences in the relapse rate. There was a significant difference between the two groups (P = 0.013).

The results demonstrated statistically significant differences in remission (P = 0.001) and relapse (P = 0.013) rates (Table 4) between the two groups at 48 weeks. The T group exhibited remission rates of 73.33, 76.66, and 66.66% at 12, 24, and 48 weeks, respectively while the T + P group achieved remission rates of 81.66, 86.66, and 91.66%, respectively for the same time periods. The T + P group achieved higher remission rates and lower relapse rates than the T group. In the subgroup analysis, the T + P group retained a high remission rate and a low relapse rate, and these between-group differences were statistically significant. The mean remission times were 20.33 ± 2.75 weeks and 9.50 ± 1.81 weeks in the T and T + P groups, respectively. At 48 weeks, 13 patients in the T group and 3 in the T + P group experienced relapses. Kaplan–Meier curves were used to illustrate differences in remission (Fig. 3a) and relapse (Fig. 3b) rates between the two groups.

**Table 4:** Remission rates of the two study groups.

Time		T (n = 60)	T + P (n = 60)	χ2	P value
12 weeks	CR	3	11	3.962	0.047
	PR	41	38		
	Relapse	5	5	0	1
	Remission rate (%)	73.33	81.66	0.054	0.817
24 weeks	CR	9	24	9.404	0.002
	PR	37	28		
	Relapse	5	5	0	1
	Remission rate (%)	76.66	86.66	2.004	0.157
48 weeks	CR	15	25	4.024	0.045
	PR	25	30		
	Relapse	13	3	11.368	0.013
	Remission rate (%)	66.66	91.66	5.841	0.001

COX analysis was conducted on the matched data. Univariate COX regression analysis of remission showed that the baseline anti-PLA2Rab levels, therapy, and blood glucose levels may be related to remission (Table 5). Significant variables in univariate analysis were included in multivariate analysis. For predicting remission, Multivariate COX regression analysis revealed that anti-PLA2Rab was positive at baseline may be an independent risk factor and therapy of T + P may be an independent protect factor (Table 5). Furthermore, anti-PLA2Rab negativity at 12 weeks was not an independent risk factor for remission.

**Table 5:** Univariate and multivariate COX regression analysis of the remission outcome.

Characteristic	Univariate				Multivariate			
	HR	95% CI		P value	HR	95% CI		P value
Gender (male/female)	0.687	0.458	1.030	0.069				
Age (years old)	0.997	0.983	1.011	0.657				
SBP (mmHg)	1.000	0.988	1.012	0.982				
DBP (mmHg)	1.003	0.990	1.016	0.667				
Hb (g/L)	0.995	0.984	1.007	0.450				
ALB (g/L)	1.036	0.989	1.086	0.135				
Scr (umol/L)	0.994	0.982	1.007	0.399				
eGFR (mL/min/1.73 m^2^)	1.004	0.992	1.016	0.530				
PLA2R(Ru/mL)	0.998	0.996	1.000	0.116				
TC (mmol/L)	0.962	0.904	1.024	0.228				
TG (mmol/L)	1.010	0.900	1.133	0.869				
Blood-glucose (mmol/L)	0.735	0.540	0.999	0.049				
24-h urine proteinuria (g/24 h)	0.951	0.881	1.025	0.189				
Anti-PLA2R ab ( +)	2.289	1.250	4.189	0.007	2.065	1.118	3.814	0.020
3 months anti-PLA2R ab negativization	0.735	0.491	1.101	0.135				
Threapy	0.565	0.374	0.854	0.007	0.583	0.384	0.885	0.011

### Complications

In the follow-up period, statistical analyses were performed to examine the incidence of various complications, including infections, gastrointestinal reactions, hepatotoxicity, hyperglycemia, new-onset hypertension, and osteoporosis. Five patients in the T + P group were diagnosed with osteoporosis, but this complication was not observed in the T group. However, the difference between the groups was not statistically significant (as shown in Table 6). This may be attributable to the use of prednisone, which we administered to the patients along with calcium to prevent osteoporosis.

**Table 6:** Side effects in patients in two groups.

	T(n = 60)	T + P(n = 60)	χ2	P value
Infection	10(16.6%)	9(15%)	0.063	0.803
Gastrointestinal reaction	5(8.3%)	7(11.6%)	0.37	0.543
Hepatotoxicity	2(3.3%)	3(5.0%)	0	1
Hyperglycemia	9(15.0%)	11(18.3%)	0.24	0.624
Osteoporosis	0	4(6.6%)		0.119
New-onset hypertension	3(5.0%)	2(3.3%)	0.0001	1

## Discussion

A study on the recurrence and remission of MN treated with TAC revealed that the remission rate was 84% at 18 months^13^. Another study reported that TAC and CTX combined with corticosteroids in treatment of MN, both had remission rates > 80%, but only the TAC group experienced relapse^21^. Liang et al. reported that 182 patients with TAC showed a cumulative PR or CR of 31, 57, and 75% at 6, 12, and 18 months, with a relapse rate of 36.4%^22^. Ramachandran et al. evaluated TAC combined with corticosteroids and found remission rates of 74 and 71% at 6 and 12 months^23^. The lower remission rate may be related to patient resistance. The results of these studies are presented in S Table 5. Our study revealed that at the 48-week follow-up, 24 patients in the T + P group experienced CR, and 31 achieved PR; the total response rate was 91.66% in the T + P group and 66.66% in the T group. The study had a low remission rate in the T group, which may be related to the duration of follow-up and higher relapse rate. Compared with treatment with tacrolimus alone, tacrolimus with low-dose prednisone improved the remission rate and reduced the relapse rate in patients with MN. In addition, there was no difference in the side effects of low-dose prednisone. Treatment with tacrolimus and low-dose prednisone considerably improved remission rates. The difference in remission rates between the two groups cannot exclude the effects of low-dose prednisone.

As a macrolide lactone antibiotic with potent immunomodulatory properties, TAC effectively inhibits T lymphocytes, and prevents B lymphocyte mitogenesis, as well as proteinuria caused by glomerular diseases; most podocytes are diseased^24–26^. TAC reduced podocyte apoptosis and inhibited the damaging effects of angiotensin II on podocytes^27^. Peng et al. demonstrated that TAC treatment reduces glomerular angiopoietin-like 4, glomerular immune deposits, and circulating IgG levels, decreases proteinuria, and promotes podocyte repair^28^. Additionally, prednisone protects podocytes by inhibiting the FAK/RANKL/MAPK signaling pathway (FAK/RANKL/MAPK) in kidney tissue^29^. A previous study^1^ revealed that TAC increased the ALB levels in patients at a faster rate than it reduced their urinary protein levels. The underlying mechanism includes reducing FK506-mediated LDH levels and preventing IL-6-induced suppression of ALB synthesis^30^. Migita et al.^31^ demonstrated that TAC enhanced dexamethasone-induced apoptosis of T cells in vivo and in vitro, and further showed that this interaction might enhance the therapeutic immunosuppression achieved by TAC and corticosteroids. Dual therapy with glucocorticoids and TAC enhances the role of steroids by increasing their affinity for the glucocorticoid autoreceptors^32^. In addition, another study showed that corticosteroids influence TAC levels within the first six months of immunosuppressive therapy in kidney transplant recipients^33^. We hypothesized that dual therapy with TAC and prednisone might reduce the recurrence rate of MN because both drugs protect podocytes from apoptosis. This may be because corticosteroids and TAC are CYP3A and P-glycoprotein 1 substrates^34^, which cause immunosuppression via T cell apoptosis^35^. Our findings are in agreement with the results of these studies.

MN recurrence may be related to circulating anti-PLA2R antibodies that bind to PLA2R antigens^26^. One study showed that patients with immunological remission preceded those with clinical remission by months^36^, and another study showed revealed that some patients exhibited clinical-serological dissociation^37^. Previous studies have shown that serum anti-PLA2R Ab titers may indicate severe clinical manifestations and that antibodies reflect clinical disease activity^19,20^. The subgroup analysis in this study showed more severe proteinuria when the serum anti-PLA2R antibody was positive, and at 48 weeks, the anti-PLA2R Ab, urinary protein, and relapse rates were higher. In the present study, the T + P group showed higher remission rates and lower relapse rates when the anti-PLA2R Ab was positive (S Table 4). Most patients in this study developed clinical remission 2 or 3 months after immunologic remission, which was accompanied by an elevation of antibodies at relapse. Stefan et al. found that negativity for anti-PLA2R antibodies at three months was an independent predictor of remission^38^; however, in our study, this was not observed. We believe that this difference may be related to regional differences or the sample size. Another study reported that anti-PLA2Rab levels might be an independent risk factor for predicting remission in patients with MN^39^, which was also confirmed in the current study.

Relapse is the determining factor for MN treatment. Caro et al.^13^ showed that almost half of the patients (44%) experienced relapse, which may be associated with TAC discontinuation. They also showed that recurrence may be associated with the duration of the full tacrolimus dose. Previous studies investigating the use of prednisone in nephrotic have indicated that a longer duration of therapy is important to reduce the risk of relapse^40^. However, renal toxicity and infections are the major concerns associated with this approach. At the end of this study follow-up, the mean duration of therapy with tacrolimus or with tacrolimus and prednisone was 36.2 ± 3.4 and 30 ± 1.2weeks. The relapse rate was significantly reduced with combination with a low dose of prednisone. The relapse rate of 21.66% in the T group was significantly higher than that 5.0% in the T + P group at 48 weeks. During follow-up, three patients relapsed more than twice in the T group, and one patient received rituximab for the third relapse.

TAC, a calcineurin inhibitor, induces renal toxicity. A previous study has shown that long-term and high-dose oral TAC causes calcineurin inhibitor-related nephrotoxicity^30^. Acute renal failure was not observed in any patient during follow-up. Although the Scr levels gradually increased, no significant differences were observed between the groups at baseline. In this study, the serum concentration of TAC was maintained between 3 and 10 ng/mL, which was higher than that reported in previous trials^41^. This indicates that an appropriate TAC dose may reduce calcineurin inhibitor-related nephrotoxicity. Some patients in the T + P group developed osteoporosis and were administered oral calcium supplements. Nonetheless, our findings revealed that clinicians should focus on the early detection and treatment of MN. Eleven patients in the dual therapy group developed hyperglycemia, which was effectively treated by reducing the prednisone dose. There were no significant differences in gastrointestinal reactions, hepatotoxicity, or new-onset hypertension between the T and T + P groups^11^.

This study had some limitations. First, all patients were treated at the same center. Secondly, the study design was non-blinded, non-randomized, non-controlled, and short-term. Thirdly, our study mainly included moderate-risk PMN, the result showed that Tacrolimus plus prednisolone combination better than Tacrolimus alone can be considered only mild to moderate PMN as the study excluded severe PMN (with massive proteinuria and renal insufficiency). Therefore, a randomized controlled trial is required to verify the validity of these treatment regimens.

In conclusion, our study revealed that TAC and low-dose prednisone dual therapy was significantly efficacious in managing MN and lowered the recurrence rate. Long-term therapy with TAC and low-dose prednisone reduced the risk of relapse. Anti-PLA2R Abs may be associated with the course and prognosis of primary membranous nephropathy. Monitoring anti-PLA2Rab may be critical in the treatment of membranous nephropathy.

### Supplementary Information

Supplementary Tables.

## Data Availability

The raw data in this study are available from the corresponding author upon reasonable request.
