# Supplementary material for: Comparison of tacrolimus with or without prednisone therapy in primary membranous nephropathy: a retrospective clinical study
Source: Sci Rep. 2024 Jun 20;14:14214. doi: 10.1038/s41598-024-64661-w (PMC11190188; doi:10.1038/s41598-024-64661-w)
Supplement: Supplementary file 1 — Supplementary Tables. [file 41598_2024_64661_MOESM1_ESM.docx]

**S table 1**

Total renal chronicity score

| The scores of GS(percentage of glomeruli with global sclerosis and ischemic glomeruli: 0, <10% 1, 10-25% 2, 26-50% 3, >50%), TA(percentage of renal cortex invol ved: 0, <10% 1, 10-25% 2, 26-50% 3, >50%), IF(percentage of renal cortex invol ved: 0, <10% 1, 10-25% 2, 26-50% 3, >50% ) and arteriosclerosis(extent of thickening of the intima: 0, intimal thickening < thickness of media 1, intimal thickening ≥ thickness o f media) are added to grade into | |
| --- | --- |
| **minimal (0–1 total score)** | **mild (2–4 total score)** |
| **moderate (5–7 total score)** | **severe (≥ 8 total score)** |

**S table 2 Characteristics of patients in the two subgroup groups.**

| Characteristics | anti-PLA2R ab(+) | anti-PLA2R ab(-) | Z/χ2 | P value |  |
| --- | --- | --- | --- | --- | --- |
| Gender (male/female) | 56/51 | 7/6 | 0.011 | 0.918 |  |
| Age (years old) | 47(38,55) | 45(35.5,64) | -0.194 | 0.846 |  |
| SBP (mmHg) | 130(120,138) | 125(119,130) | -1.794 | 0.073 |  |
| DBP (mmHg) | 81(75,90) | 82(79,90) | -0.537 | 0.591 |  |
| Hb (g/L) | 128(114,139) | 135(131,144) | -1.597 | 0.110 |  |
| ALB (g/L) | 25.4(22.3,28.1) | 28(25.3,29.2) | -1.862 | 0.063 |  |
| Scr (umol/L) | 63(55,76) | 64(56,72) | -0.182 | 0.856 |  |
| eGFR (mL/min/1.73㎡) | 105.1(96.1,113.3) | 102.4(92.7,116.8) | -0.270 | 0.787 |  |
| TC (mmol/L) | 6.8(5.4,9.0) | 7.0(6.0,8.8) | -0.473 | 0.636 |  |
| TG (mmol/L) | 2.0(1.3,3.0) | 2.5(1.0,3.2) | -1.188 | 0.235 |  |
| Blood-glucose (mmol/L) | 4.5(4.2,4.8) | 4.5(3.8,5.1) | -0.413 | 0.679 |  |
| 24-hour urine proteinuria (g/24h) | 4.5(3.6,6.3) | 3.6(3.5,3.8) | -2.850 | 0.004 |  |
| Chronicity score | 2.0(1.0,3.0) | 1.0(0.5,1.5) | -3.159 | 0.002 |  |

**Stable 3 Remission rates of the subgroups.**

| Time | High titer (N=34) Non-high titer (N=86) | | | | | | | | |
| --- | --- | --- | --- | --- | --- | --- | --- | --- | --- |
|  |  | T | T+P | χ2 | P Value | T | T+P | χ2 | P  Value |
| 12weeks | CR | 0 | 1 |  |  | 3 | 10 |  |  |
|  | PR | 8 | 8 |  |  | 33 | 30 |  |  |
|  | Relapse | 1 | 2 |  | 0.571 | 4 | 3 | 0.017 | 0.898 |
|  | Remission rate(%) | 42.1 | 60.0 |  | 0.491 | 87.8 | 88.8 | 0.499 | 0.480 |
| 24weeks | CR | 0 | 4 |  |  | 9 | 20 |  |  |
|  | PR | 8 | 6 |  |  | 29 | 22 |  |  |
|  | Relapse | 2 | 2 |  | 1 | 3 | 4 | 0 | 1 |
|  | Remission rate(%) | 42.1 | 66.6 |  | 0.185 | 92.6 | 93.3 | 0 | 1 |
| 48weeks | CR | 0 | 5 |  |  | 15 | 20 |  |  |
|  | PR | 5 | 6 |  |  | 20 | 24 |  |  |
|  | Relapse | 7 | 1 |  | 0.053 | 6 | 2 | 1.571 | 0.210 |
|  | Remission rate(%) | 26.3 | 73.33 |  | 0.014 | 85.3 | 97.7 |  | 0.088 |

**S table 4 Remission rates of the subgroups.**

| Time | Anti-PLA2R ab(+) Anti-PLA2R ab(-) | | | | | | | | |
| --- | --- | --- | --- | --- | --- | --- | --- | --- | --- |
|  |  | T  (53) | T+P (54) | χ2 | P Value | T  (7) | T+P  (6) | χ2 | P  Value |
| 12weeks | Remission rate(%) | 69.81 | 79.62 | 1.367 | 0.242 | 100 | 100 |  |  |
| 24weeks | Remission rate(%) | 73.58 | 85.18 | 2.204 | 0.138 | 100 | 100 |  |  |
| 48weeks | Remission rate(%) | 62.26 | 90.74 | 12.114 | 0.001 | 100 | 100 |  |  |

**S table 5 Studies conducted on membranous nephropathy using tacrolimus.**

| **Study** | **Country** | **eGFR** | **Proteinuria** | **ALB** | **Age** | **Male%** | **Follow-up time** | **Percentage of response to therapy** |
| --- | --- | --- | --- | --- | --- | --- | --- | --- |
| Caro et al.^13^ | Spanish | 84.1±29.8 | 8.27±4.05 | 25.5±0.65 | 48±13 | 71.3 | 30（14-66） | 6months 60% |
|  |  |  |  |  |  |  |  | 12months 78% |
|  |  |  |  |  |  |  |  | 18months 84% |
| Liang et al.^21^ | China | 93.62±1.7 | 5.9±2.7 | 26.5±6.2 | 48.2±13.5 | 53.3 | 10(0.2-18) | 6months 76.7% |
|  |  |  |  |  |  |  |  | 12months 80% |
|  |  |  |  |  |  |  |  | - |
| Liang et al.^22^ | China | 98.68±20.55 | 4.67(3.22-6.55) | 25.89±5.76 | 46(32-56) | 63.7 | 27.3(19.3-41.6) | 6months 31.3% |
|  |  |  |  |  |  |  |  | 12months 57.5% |
|  |  |  |  |  |  |  |  | 18months 75.8% |
| Ramachandran et al.^23^ | India | 96.72±27.13 | 6.76±3.59 | 20.2±0.67 | 38.66±1.91 | 77.1 | 12 | 6months 74% |
|  |  |  |  |  |  |  |  | 12months 71% |
|  |  |  |  |  |  |  |  | - |
